# Outcomes From an Interprofessional Geriatric Outreach and Training Program

**DOI:** 10.1093/geroni/igac044

**Published:** 2022-07-01

**Authors:** Anna-Rae Montano, Juliette Shellman, Millicent Malcolm

**Affiliations:** School of Public Health, Brown University, Providence, Rhode Island, USA; Center of Innovation in Long Term Services and Supports, Providence VA Medical Center, Providence, Rhode Island, USA; School of Nursing, University of Connecticut, Storrs, Connecticut, USA; School of Nursing, University of Connecticut, Storrs, Connecticut, USA

**Keywords:** Education, Home- and community-based services, Primary care, Training

## Abstract

**Background and Objectives:**

Interprofessional education (IPE) is necessary to train health care professionals to work collaboratively for the care of older adults. Geriatric Outreach and Training with Care! (GOT Care!) was an innovative academic training program designed to provide an IPE opportunity for health care students and faculty while providing care to community-dwelling older adults. The objectives of this program evaluation were to: (a) examine students’, older adult participants’, and primary care providers’ (PCPs) perceptions toward their participation GOT Care! and (b) examine patient outcomes to identify program strengths and areas for improvement.

**Research Design and Methods:**

Formative and summative program evaluation methods were utilized to evaluate student, older adult participant, and provider perceptions of participating in GOT Care!. A total of 221 pharmacy, physical therapy, nursing, social work, medicine, and public health students from a single public university in northeastern United States, 38 community-dwelling older adults, and 33 PCPs were included. Means, standard deviations, and percentages were computed for survey data. The contextual data gathered from interviews and open-ended questions were analyzed using Borkan’s immersion–crystallization approach to generate themes.

**Results:**

Overall, the students, older adults, and PCPs appreciated GOT Care!. Students reported learning about the unique challenges to geriatric care and how to communicate with other professionals. The older adults appreciated the thorough interprofessional assessment and that the students could learn from them. The PCPs noted the unique insights into their patients’ health that would not present at a typical office visit.

**Discussion and Implications:**

GOT Care! leveraged academic and community partnerships to provide an IPE opportunity and care to vulnerable older adults. Positive outcomes such as older adult, student, and PCP satisfaction, and a reduction in emergency department visits support ongoing utilization and evaluation of these IPE programs.

**Translational Significance:** Geriatric Outreach and Training with Care! aimed to address a workforce shortage of clinicians prepared with geriatric and interprofessional competencies as well as provide clinical care to community-dwelling older adults. The older adult participants appreciated the interprofessional assessments, while the student and primary care provider participants learned about the unique challenges and advantages to interprofessional geriatric care. A reduction in emergency department visits and an increase in referrals to community services were observed. Interprofessional geriatric training programs that integrate existing community-based services will be essential in preparing the health care workforce.

## Background

By 2034 the number of people 65 years and older is projected to climb to 77.0 million (U.S. Census Bureau, 2019). Older adults are best served by the coordinated efforts of an interprofessional collaborative practice (IPCP) team to efficiently and effectively address the complexity of their health care needs. Unfortunately, inadequate numbers of health care professionals are prepared to deliver this type of care (IOM, 2008; Partnership for Health in Aging, 2014). The National Center for Interprofessional Practice and Education (2016) and the World Health Organization (Gilbert et al., 2010) cite the importance of IPCP team care toward improvements in quality of care and patient outcomes with reduced costs. While a number of studies have evaluated the responses of students to interprofessional education (IPE), there is a lack of data on the impact of IPE programs from patient and primary care provider (PCP) perspectives. This paper describes results from all three groups.

### Interprofessional Education

Early involvement of students in IPCP teams promotes best practice in clinical settings. A systematic review and meta-analysis by Guraya and Barr (2018) found that pre–post evaluations of IPE courses demonstrated improved attitudes, knowledge, and skills for learners regarding collaborative teamwork. Challenges to IPE that were identified include limited resources, time, and course space for the growing number of health care students required to enroll in IPE (Guraya & Barr, 2018). IPE opportunities allow students to learn from other disciplines to enhance geriatric care while also positively affecting student attitudes toward interprofessional collaboration (Conti et al., 2016; Dockter et al., 2020; Gould et al., 2015; Heflin et al., 2014). Lee et al. (2013) described a service-learning IPE program involving nursing, pharmacy, and nutrition students that provided a comprehensive in-home assessment for a vulnerable group of older adults along with recommendations and a follow-up plan. The assessments identified older adults at risk for malnutrition and polypharmacy. Using their assessment data, students worked in interprofessional groups to create a plan for follow-up and conduct home visits to implement the plan with the older adults. Student reflections indicated high satisfaction with the experience and reported feeling they could make a difference with an underserved community because of IPE. Similarly, a two-staged sequential mixed methods study conducted among 282 medical, dental, pharmacy, and health sciences students assessed the perspectives of students toward IPE and collaborative practice using the Readiness for Interprofessional Learning Scale (RIPLS). Students showed readiness to adopt IPE with high median scores on the RIPLS and positive attitudes toward IPE (Sulaiman et al., 2021).

Despite the recent successes of interprofessional geriatric education in preparing the workforce, barriers to training program implementation are reported including: (a) previous negative attitudes and experience toward interprofessional geriatric training and care, (b) academic constraints, (c) insufficiently trained educators, (d) scheduling challenges, and (e) underwhelming interest of the students in working with older adults (Abu-Rish et al. 2012; Bardach & Rowles, 2012). Key facilitators to successful IPE are reported as: (a) administrative support, (b) staff support, (c) funding, (d) leadership buy-in, and (e) student support (Abu-Rish et al., 2012; Graybeal et al., 2010). In this study, we addressed the need to evaluate the responses of student, older adult participant, and PCP perceptions of participating in an interprofessional outreach and training program: Geriatric Outreach and Training with Care! (GOT Care!).

### GOT Care!

A well-trained workforce in the specialty of geriatrics is necessary to address the need for older adults to access high-quality care. GOT Care! provided innovative didactic and clinical training with IPCP teams of students and faculty. This model brought together an academic and health system partnership with an interprofessional faculty team of geriatric experts from nursing, dental medicine, public health, pharmacy, medicine, physical therapy, and social work. Students from each of these disciplines experienced unique hands-on opportunities to learn together in the classroom. The students and faculty conducted home visits for older adults with multiple chronic conditions and high emergency department (ED) use. A unique feature of GOT Care! is that the faculty–student IPCP teams provided in-home comprehensive geriatric assessments and collaborated with PCPs to reduce the older adults’ risks for hospitalization. While bringing a team of health care providers into a home may seem cumbersome, the home provides unmatched opportunities for assessment of the environment, family interactions, and socioeconomic factors that can affect the older adult’s health and well-being. The IPCP team visits consisted of the following; (a) an initial intake assessment conducted by pharmacy faculty, the Nurse Navigator (home health care nurse), and the graduate assistant data collector, (b) the interprofessional student–faculty teams made two home visits to conduct the comprehensive geriatric assessment, and (c) one final follow-up visit with the community-dwelling older adult to review the recommendations including referrals (Malcolm et al., 2017).

### Conceptual Framework

**Figure 1.**
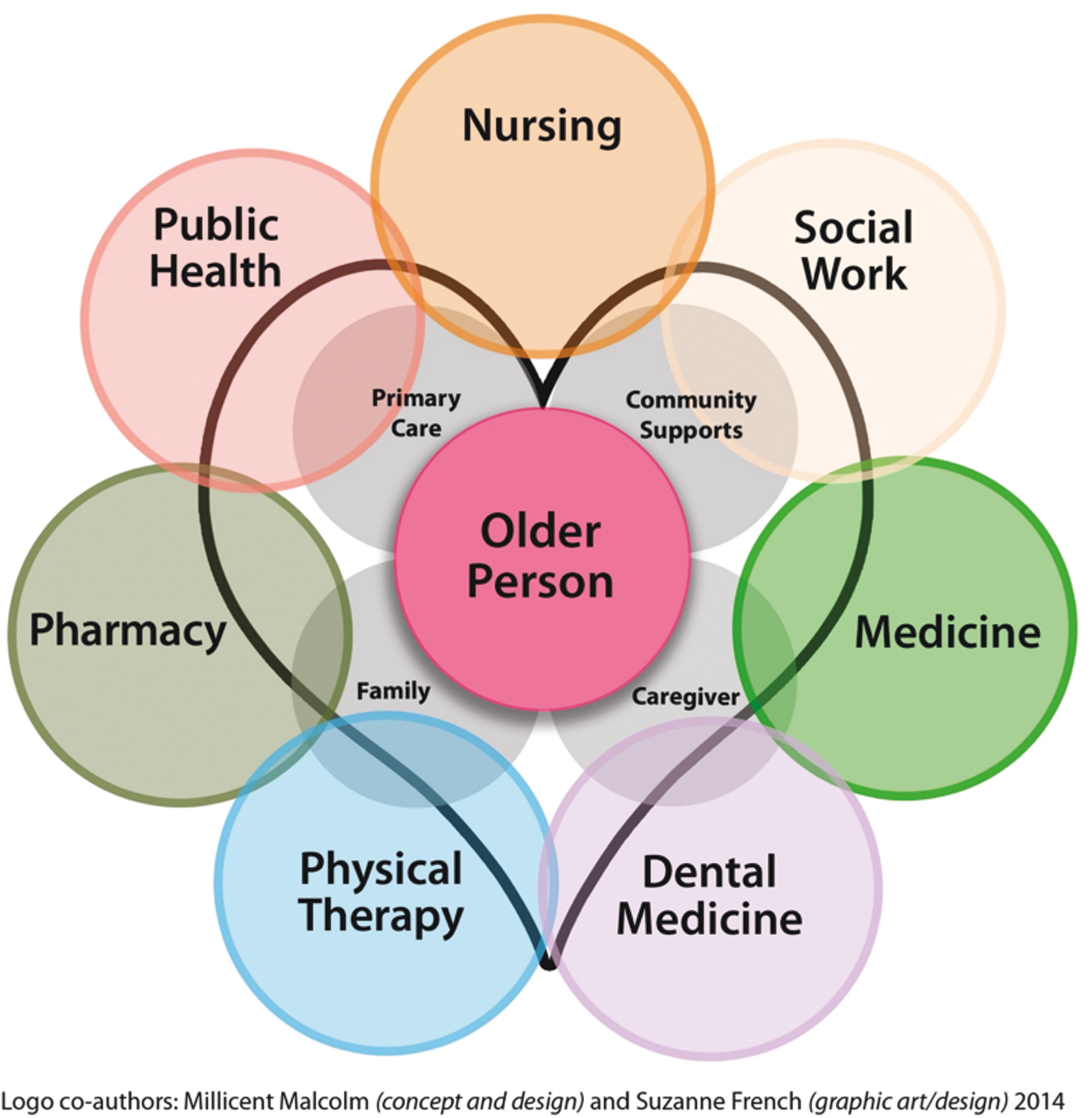
Geriatric Outreach and Training with Care! (GOT Care!) interprofessional collaborative practice model.

The Interprofessional Education for Collaborative Patient-centered Practice Model (D’Amour & Oandasan, 2005) provided the underpinnings for GOT Care! development and implementation. The GOT Care! Interprofessional Practice Model (Malcolm, 2014) provided the structure for the program, with the goal of improved coordination of and access to care among older adults. Figure 1 illustrates the central components of the model. The model reflects the overall synchronization and shared efforts of a well-trained IPCP health care team that connects an older adult and their support system. Key to the success of the model was harmonious collaboration among the professionals with the shared goals of preparing and enhancing the geriatric health care workforce while improving health outcomes for the older adults.

### GOT Care! Program Evaluation

The objectives of this program evaluation were to: (a) examine students’, older adults’, and PCPs’ perceptions toward their participation in GOT Care! and (b) examine older adult participant outcomes to identify program strengths and areas in need of improvement. Data collection took place during the first 2 years of program implementation. The specific aims that guided this evaluation were as follows:

1. To determine students’, older adults’, and provider perceptions toward their participation in GOT Care!.
2. To monitor community service referrals and ED visits among a cohort of GOT Care! older adults during the first 2 years of implementation to identify program strengths and areas needing improvement.

### Setting

GOT Care! took place in an urban area in the northeast United States. A community needs assessment revealed a rapidly growing older adult population with excessive ED use at rates higher than the state. Both the 65+ and 85+ population groups were noted to be relying on the ED for ambulatory sensitive conditions not expected to end in hospitalizations (University of New England, 2008). This aggregate of older adults was serviced by a large community hospital system, which included various departments and services including Community Benefits Services, Hospital Home Care, Center for Behavioral Health, and the Center for Chronic Disease Management.

## Method

### Design

Formative and summative program evaluation methods were utilized to evaluate student, older adult participant, and provider perceptions of participating in GOT Care!. The Internal Review Board of the university and the hospital provided a quality improvement designation for this project.

### Data Collection

Participant observations, surveys, and open-ended questionnaires were utilized to gather data regarding students’ (*N* = 221), older adults’ (*N* = 38), and PCPs’ (*N* = 33) perceptions of participating in GOT Care!. Data collectors included the GOT Care! team members observations of the training and outreach experience and a trained graduate research assistant for administration of older adult participant and student satisfaction surveys pre- and post-GOT Care! experience. PCPs were mailed a survey at the end of the experience. Responses were mailed back directly to the program evaluator. Data regarding participant ED visits and community referrals were collected from participants’ electronic medical records.

### Data Analysis

Demographic characteristics and descriptive data were analyzed using SPSS 20. Means, standard deviations (*SD*s), and percentages were computed for survey data. The contextual data gathered from interviews and open-ended questions were analyzed using an immersion–crystallization approach developed by Borkan (1999). The steps in this process included: (a) initial engagement with the topic, (b) crystallization, (c) immersion and illumination from the data texts, (d) explication and synthesis, (e) consideration of alternative interpretations, and (f) reporting the account. The GOT Care! team selected this analysis because immersion–crystallization occurs before data collection, during the study design process, at the beginning and end of data collection, and during the reporting step. The continuous analytic technique developed by Borkan (1999) fits well with the specific aims of this study.

## Results

### Sample

A total of 221 students from a single public university, 38 older adult participants, and 33 PCPs were included in this program evaluation. Table 1 presents data on students and their respective professions. Approximately 50% of the sample were nursing students. The average age of the older adults was 81.04, (*SD* = 7.37). Additional demographic data indicate that 70% of the older adult participant sample was female, 83% were White, 58% reported high school as their highest level of education, and 50% of the participants reported having one or more chronic illnesses. The majority of the PCPs practiced within the community hospital health care system and 100% of the PCPs had at least one of their patients in the GOT Care! program.

**Table 1.** Student Participant Professions

Student professions	Cohort 1 (*n* = 60)	Cohort 2 (*n* = 69)	Cohort 3(*n* = 48)	Cohort 4 (*n* = 44)	Total (*N* = 221)
Dental	12	23	0	0	35
Nursing					
Undergraduate	14	7	13	16	50
Graduate	12	15	15	12	54
Medicine	1	5	5	1	12
Pharmacy	11	10	8	5	34
Physical therapy	7	6	6	7	26
Public health	0	1	0	1	2
Social work	3	2	1	2	8

The immersion–crystallization approach to data analysis revealed many insights into student, older adult participant, and provider perceptions regarding participation in the GOT Care! training and outreach program. The overall feedback from the three groups revealed positive experiences as demonstrated in the following themes.

### Student Perceptions

Students answered open-ended questions regarding their experience of conducting comprehensive geriatric assessments in the home and participating in IPCP team care. The following themes emerged from the data: (a) *collaboration and problem-solving with other professions*, (b) *learning unique challenges of geriatric care*, and (c) *preference for hands-on interprofessional learning*.

#### Theme 1: collaboration and problem-solving with other professions

Students repeatedly commented that the GOT Care! team demonstrated “open communication” and created an atmosphere where they felt “free to express opinions and patient concerns.” Students also recognized that within an IPCP team, members may have different priorities and this may be challenging to work through during case conferences. However, students commented they felt that each opinion was weighed equally during the process, which resulted in a positive learning environment and better care for the older adult participant. For example, one student commented, “by working together we were able to identify and address more problems than if we had each worked independently. Each member of the team was allowed to be involved in the patient care.” The data revealed that the interprofessional interactions during the home visits and IPCP case conferences were helpful in their learning how to communicate with other health professionals. For example, one participant noted, “I’ve learned a lot about roles/responsibilities and I feel like I know a lot more about how to communicate and problem solve with different specialties.”

#### Theme 2: learning unique challenges of geriatric care

The experiences of making home visits opened the students’ eyes to the complexities of caring for older adults in the home. The findings revealed the challenges of providing home care such as environmental hazards, family members with caregiver burden, and needing to be able to think “on your feet” to adapt to the home setting. In another example, the interprofessional approach to the home visit enabled students to see that psychosocial aspects of patient care are just as important as the medical aspects of care. Students also expressed disbelief about the number of medications issues the geriatric assessment uncovered. Overall, students reported they enjoyed being part of the team home visits to see other disciplines in action while improving patient outcomes. Positive comments included, “GOT Care! is a very positive approach to patient care” and “being able to work with a variety of students to see how each profession approaches a problem was very useful.”

#### Theme 3: preference for hands-on interprofessional learning

One key finding during the first semester of implementation was the students’ preference for hands-on interprofessional learning. This insight was described in open-ended questions and through observations made by faculty during the pre- and postconferences. For example, one student stated, “because the group is so big, many of the students aren’t able to be so engaged in the hands-on process or report out at case conferences.” Faculty also observed that not all students were participating during the postconference when problem-solving and setting priorities of the recommendations for the PCPs. Table 2 displays an example of the immersion–crystallization analysis that led the team to this insight.

**Table 2.** Examples of Insights Revealed Using Immersion–Crystallization Technique

Stage of immersion–crystallization approach	Process	Example of insight
Initial engagement	Discussion of biases with data collectors	Bias toward program success
Describing	Debriefing sessions with data collectors	Participants describing need for more hands-on learning in surveys and in journals
Crystallization	Insights gained during pre- and postconferences.	Faculty report not all students participating in discussions
Immersion/illumination	Repeated review of data	Initial survey data review led to more in-depth discussions with faculty, data collectors
Consideration of alternative interpretations	Discussion of insights with data collectors	Evaluator and project director discuss findings
Reporting	Presentation of results Discussion of findings with Geriatric Outreach and Training with Care! team Manuscript development	Project director and faculty revise home visit and postconference to include more student involvement and hands-on activities Poster and paper presentations at local and national conferences.

Students repeatedly made positive comments about the interprofessional home visits and the opportunities these visits provided for learning about the other health professions. Several students commented on the need to increase opportunities for interprofessional learning. One student stated, “I would appreciate more opportunities to do home visits with different disciplines. I followed social work and pharmacy, but I wanted to follow physical therapy and the others, too.”

### Patient Perceptions

The GOT Care! team made home visits to 44 older adult participants during the first 2 years of program implementation. Two older adults with chronic psychiatric illnesses dropped out after the initial assessment, three other older adult participants did not complete the postexperience surveys because they were hospitalized during program implementation, and one older adult participant was unavailable for follow-up data collection. The final program evaluation sample (*N* = 38) completed pre- and postexperience survey interviews.

The following three themes emerged from the survey and interview data collected from the GOT Care! older adult participants: (a) highly satisfied with care, (b) “Its wonderful students can learn from us,” and (c) taking the time to understand me.

#### Theme 1: highly satisfied with care

Overall, the patients (*N* = 38) expressed high satisfaction with the GOT Care! team as evidenced by 100% of the patients reporting that they were treated with courtesy and respect by the team. The majority of participants (88%) reported that the GOT Care! team always explained things in a way that was easy to understand. Ninety-four percent of the patients felt the GOT Care! team always listened carefully to them.

During the end of the experience interviews the patients repeatedly expressed how much they liked being part of GOT Care!. For example, one participant stated, “I couldn’t say the program can get any better than it is now.” The patients felt GOT Care! was “very meaningful” and that the students were “ambitious.”

#### Theme 2: “Its wonderful students can learn from us”

While one of the goals of the GOT Care! team was to improve patient outcomes, one unexpected finding was the participants’ perspectives that they were helping to teach the students by participating in GOT Care!. For example, one patient reported, “I’m very satisfied with the GOT Care! program and it is wonderful students can learn from us,” while another stated, “it would be nice if the students could come more often.”

#### Theme 3: taking the time to understand me

Initially, the team was concerned with the length of time needed to implement the comprehensive geriatric assessment as well as the size of the IPCP team making the home visit. The participants, however, were appreciative of the interprofessional approach and the thorough assessment conducted during the visit. Comments such as, “I’m glad they came here and took the time to ask me those questions” and “it’s very useful to air out our issues and that someone recognizes the problems we are confronted with” reflect that the participants appreciated the time the team took to conduct the comprehensive geriatric assessments.

### PCP Perceptions

Overall the PCPs expressed satisfaction with the GOT Care! team. It is important to note that the PCP participant survey response rate increased from 40% at the start of the program to 100% by the last semester. Table 3 identifies means and *SD*s of provider perceptions of working with the GOT Care! team.

**Table 3.** Primary Care Providers’ Perceptions of Interaction With the Interprofessional Collaborative Practice Team

Communication and information exchange items	*N*	Min	Max	*M*	*SD*
The GOT Care! team provided relevant suggestions for my patient.	33	5	7	6.4	.69
The GOT Care! team comprehensive geriatric assessment may help adverse events including emergency room visits.	33	5	7	6.0	.81
I would not hesitate to recommend other frail patients to the GOT Care! team	33	6	7	6.7	.48
After reviewing information from the GOT Care! interprofessional team, I am more likely to use an interprofessional approach to manage my frail patients in the future.	33	6	7	6.6	.51

*Notes*: GOT Care! = Geriatric Outreach and Training with Care!. Based on Likert scale (“Strongly Disagree” = 1 to “Strongly Agree” = 7).

Contextual data revealed that the PCPs perceived GOT Care! as a more cohesive approach to patient care, citing the team provides them with insight into patient problems such as medication issues, living arrangements, and need for direct family interventions that cannot be identified through a typical office visit.

### Older Adult Participant Outcomes

One hundred and fifty home visits were made over the 2-year data collection period. Forty-six comprehensive geriatric assessments were conducted by the GOT Care! team. As a result of the home visits and assessments, the team made numerous referrals to different community services. For example, 19 older adult participants were referred to the hospital system’s home care agency and seven patients were referred to the Center for Behavioral Health for further assessment and monitoring. Other referrals were made to dental services (*n* = 5), social work (*n* = 4), palliative care (*n* = 4), nutritional education (*n* = 5), and for chronic disease management (*n* = 6).

The number of ED visits made by the GOT Care! older adult participants was assessed by reviewing electronic medical record data preadmission to GOT Care! and post-GOT Care! participation. There was a 34% decrease in mean ED visits 18 months pre-GOT Care! compared to 18 months post-GOT Care! and ED visits for participants were significantly higher in the 18 months pre-GOT Care! compared to post-GOT Care! (Montano et al., 2020). Significant predictors of ED visits post-GOT Care! included the number of ED visits pre-GOT Care!, a diagnosis of diabetes, and polypharmacy (Montano et al., 2020).

## Discussion

The goal of GOT Care! was to create an IPE opportunity for students while providing high-quality interprofessional geriatric care. The results of this program evaluation provided insight into student, older adult participant, and PCP perceptions of their experiences participating in GOT Care!. Older adult participant outcomes provided important information regarding the IPCP outreach process by examining referrals to community services and preliminary data regarding ED use.

The students’ perceptions regarding their participation and learning interprofessional skills in GOT Care! were generally positive. These findings are supported by others (Conti et al., 2016; Dockter et al., 2020; Gould et al., 2015; Heflin et al., 2014; Sulaiman et al., 2021) and have implications for interprofessional geriatric care.

As a result of the GOT Care! experience, students perceived the need for additional interactions with other team members as a way of learning the roles of all the professions. The faculty assessed this feedback and consequently reorganized the IPCP team home visits to increase interactions. Transportation to the patient’s home was rearranged so that the IPCP teams traveled together resulting in improved interprofessional communication and increased learning regarding each other’s roles.

Patient satisfaction is an important determinant of quality of interprofessional care for older adults (Tsakitzidis et al., 2016). In this evaluation, we found that older adult participants rated the IPCP team experience very positively. Particularly, the quality of the interaction with the team such as listening to their needs, being polite and caring, and being treated with respect. The older adults also expressed satisfaction with the comprehensive geriatric assessment. An unexpected finding was the positive perception they felt about their role in teaching the students. Intergenerational interactions have been shown to increase self-worth and positive feelings among older adults (Springate et al., 2008). One can propose that the older adult participants’ perceived teaching role could result in a sense of self-worth and sense of purpose.

Understanding the PCPs’ perceptions of GOT Care! was critical in the process of delivering IPCP because they were key stakeholders who carried out the team’s recommendations. Findings from this evaluation revealed PCPs were highly satisfied with the team’s comprehensive assessment and recommendations to help their patients, in particular, the medication assessment, family dynamics, and living situations. These are three key areas of assessment that are not as easily accomplished in an outpatient setting with limited time for patient visits. Yet, these areas of assessment can hold the key to important primary care interventions to reduce ED visits and hospitalizations. In addition, the PCPs were highly satisfied with the functions of the IPCP team and would recommend such teams as an adjunct to the delivery of primary care.

The older adult participant outcomes reflected a number of important health care delivery issues: (a) identification of unmet physical and psychological needs, (b) identification of family stressors and conflicts that affect the well-being of the patient, and (c) potentially harmful environmental hazards that are a primary cause of accidents for older adults in the home. These findings provided support for IPCP to address the complex health needs of vulnerable older adults living at home. Utilizing the comprehensive geriatric assessment in the context of IPCP in the home enabled the team to address issues that cannot be addressed by PCPs and increased referrals to community services—thereby contributing to the reduction in ED visits for this population.

## Conclusion

GOT Care! leveraged a community–academic partnership to provide an IPE opportunity for health care students. The older adult participants appreciated the interprofessional assessments, while the student and PCP participants learned about the unique challenges and advantages to IPCP for community-dwelling older adults. A reduction in ED visits as well as an increase in referrals to community services were observed. Future areas of inquiry include long-term follow-up with older adults and PCPs to assess follow-up with referrals and adherence to recommendations, more in-depth quantitative analyses of patient outcomes, student follow-up postgraduation, and long-term impact on health profession curricula. As long-term care shifts to the community, interprofessional geriatric training programs that integrate existing community-based services will be essential in preparing the health care workforce and improving the life course of older adults.
